# The Hospital Nursing Supplement

**Published:** 1893-05-20

**Authors:** 


					The Hospital, May 20, 1893. Extra Supplement,
**
Wxt f&osyftal" liuvstntj ffltvtttt*
Being the Extea Nubsino Supplement ot "The Hospital" Newspapeb,
[Contributions for thin Supplement should be addressed to the Editor, Thh Hospital! 428, Strand, London, W.O., and shonld have the word
" Nnrsing" plainly written in left-hand top corner of the envelope.]
flews from tbe nursing
THE NURSE DOLLS.
The Nurse Dolls have accepted an invitation to the
exhibition which is to be held at the Mansion, Yictoria
Park, Keighley, next June, and they are asked to
Blackpool in September. As the proceeds of the
former are to go to the Cottage Hospital, ,it is specially
appropriate for the dolls to give their services on the
occasion. The Blackpool and Keighley Committees
have arranged to re-dress and renovate * them. How
many of the correspondents who made such kind offers
lately to help with the dolls will assist us instead to
add to The Hospital Collection of nursing uniforms ?
We want a sample of a pretty and suitable Cottage
Hospital outfit, and we shall be glad to hear from any
readers who are willing to send dolls dressed in the
following uniforms, as we wish to have the collection
made as complete as possible: Nottingham Children's
and General Hospitals, Oldbam Infirmary, Penzance
and Peterborough Infirmaries, Devon and Cornwall
Hospital at Plymouth, Ramsgate Infirmary, Rhyl
Children's Hospital, Richmond Hospital, Rochdale
Infirmary, Rotherham and Rugby Hospitals, Ryde and
Salisbury Infirmaries, Stafford General Infirmary,
Royal Hospital, Yentnor; Worcester General Infirmary,
Yictoria Hospital, Guernsey. Dolls should be sent
before June 17th, to Editor, 428, Strand, marked
" Dolls" in left hand bottom corner of address card.
TRAINED NURSING IN WORKHOUSES.
We have already referred to the memorial which the
Workhouse Infirmary Nursing [Association has ad-
dressed to the Local Government Board, and we feel
sure that our readers will cordially wish success to the
movement. The proposals submitted are certainly
excellent ones, and there ought to be no hesitation in
granting them. Proofs are not wanting that each sug-
gested reform is urgently required, for the newspapers
every week bear testimony to existing, abuses. The
following are some of the suggestions offered to the
Board. The nursing of the sick inmates by trained
nurses only, the services of paupers being confined to
scrubbing and cleaning. The provision of proper
nursing for the sick during the night. The importance
of placing a'trained superintendent nurse in those in-
firmaries where three or more nurses are employed. The
total separation [of ithe sick from the able-bodied, by
placing them in separate buildings. The careful separa-
tion of the Lock patients from the other patients, and
provision for their effective nursing. The provision of
expeditious and efficient means of calling aid, either
medical or administrative, in the night. The appoint-
ment of a few women inspectors, in addition to the
present inspectors, especially with a view to supervising
the Nursing Department in Infirmaries'and Work-
houses.
PITY THE CHILDREN I
If the rearing of children is looked upon as the
training of future citizens, surely the duty of ensuring
to them sound minds and healthy bodies must be con-
ceded. It is a pity this fact has not been instilled into
the mind of the gentleman who proposed the other
day that the person appointed to take charge of the
children's ward in a workhouse should relieve the
infirmary nurse at night. The care of children can
only be conscientiously undertaken by women who
enjoy good health and possessjeven tempers. How long
could either be maintained under such a nervous strain
as the {[one* proposed ? The poor little paupers have
evidently been grievously neglected in the hands of
certain old women, and a change of caretakers is
promised. Nearly everyone present at the meeting
agreed that a paid nurse ought to succeed the
various incapable paupers who have kept the poor
babies neither clean nor sound. Only one or two
thoughtful men advocated an active competent nurse,
one who would have a moral influence on these forlorn
little waifs. The majority of guardians, however,
selected a broken-down person who has acted as nurse
for " a great number of years" in the same institu-
tion. That she has broken down " more mentally than
physically," is hardly a cause for congratulation
to the children. No doubt it is cheaper to employ than
to pension a faithful old servant, but have the children
themselves no claims on the hearts as well as the
pockets of those who are themselves neither destitute
nor helpless P
UNWILLING DEPARTURES.
Men enter hospitals as patients from various classes
of society and suffering from every possible disease.
But they can mostly all be sorted into two divisions,
viz., those who want to stay in, and thosej;who fret to
go out. Men who voluntarily enter the wards a second
time are usually in no hurry to depart. They have
probably returned with the hope of a cure, and also
with reminiscences of having " found themselves very
comfortable" on the previous occasion. Strangely
enough, it is not always the man with the best home
who pines tolreturn to it; ofttimes'tis the poor creature
whose general surroundings are most miserable, who
gets restless under routine and wishes for his discharge.
Healthy workmen fidget and worry when first confined
to bed, entreating the doctor for leave to get up whilst
their fractured limbs are still in splints. By degrees
they settle down, and not infrequently towards the end
of the time ask to stay in another week or so because
"it's so awkward" getting about elsewhere. The
patients in medical wards are uncertain, some serious
diseases being attended with symptomatic restlessness,
and the sufferer, like a child, " does not know what he
wants." At home he craves for hospital treatment,
and when undergoing it he prays to be sent out again !
But there are certain elderly people, not very old, per-
haps, but feeble and spiritless, and, alas! " not wanted "
by any one. These men petition with pathetic
earnestness to be kept in a little longer, and they dis-
lxxii THE HOSPITAL NURSING SUPPLEMENT, Mat 20, 1893.
cover a new ailment at each visit of the doctor who
knows he ought to send them home, but finds the duty
made very hard by the sad appeal for " just a few days
more, please, sir!"
A WISE APPOINTMENT.
It is with great pleasure that we record the appoint-
ment of Miss Mowatt, who was trained at^St. Thomas's
Hospital, to the post of head nurse at Steyning
Infirmary. Miss Mowatt is not only fully trained, but
she has had most valuable experience at the large
workhouse infirmary at Birmingham during the last
four years. No one who has seen the Birmingham
Infirmary lately can fail to be impressed with the
excellent organisation of its nursing department. Miss
Mowatt's testimonials show that she has proved an able
assistant to the Matron, Miss Gibson, with whom she
has had the good fortune to work. Certainly the
appointment of Miss Mowatt at Steyning may be
looked upon as a fair proof that the question of trained
nurses for workhouse infirmaries is being wisely con-
sidered in certain parts of England.
A SERIOUS WARNING.
Pbobably no one can accurately compute the number
of persons suffering from infectious diseases who, at
some stage of the illness, make use of public convey-
ances without the drivers having any knowledge of the
fact. This disastrous proceeding is often the result of
wilful ignorance, the perpetrator shrinking from putting
preliminary questions which may obtain inconvenient
replies. A nurse at Cardiff recently conveyed a patient
with diphtheria in a cab without warning the driver,
and her defence that she did not know the disease was
notifiable did not prevent the magistrate from im-
posing a fine of ?5 and costs, or a month's imprison-
ment with hard labour. Public Bafety must be pro-
tected, but so must nurses, and it would be well to
know whether a code of rules regarding infectious
diseases exists at the Nursing Home into which the
child was taken, and if the nurse herself is thoroughly
trained and holding a responsible position.
"THE FLORENCE NIGHTINGALE PLEDGE."
Under this title the following pledge has been
recently administered to the nurses who have earned
the badge bestowed by the Farrand Training School
(U.S.A.) "We quote from the Detroit Free Press. "I
solemnly pledge myself before God and in the presence
of this assembly, to pass my life in purity, and to
practice my profession faithfully. I will abstain from
whatever is deleterious and mischievous, and will not
take or knowingly administer any harmful drug. I
will do all in my power to maintain and elevate the
standard of my profession, and will hold in confidence
all personal matters committed to my keeping, and all
family affairs coming to my knowledge in the practice
of my profession. With loyalty will I endeavour to
aid the physician in his work, and devote myself to tho
welfare of those committed to my care."
ANOTHER NEW NURSES' HOME.
At the Farrand Training School, U.SA.,the working
hours of the nurses have been reduced to eight, leaving
sixteen for Btudy, lectures, recreation, and rest. It is
anticipated that the results of this arrangement will be
beneficial to the health, of the graduates. Certificates
are given at the end of two years, and " it is intended
that each narse, before graduation, shall have care of
the sick in private families," says the Detroit Free Press.
In other words, the nurses are to attend a certain num-
ber of private cases in the course of their two years'
training. A beautiful Home for the staff is now com-
pleted, and numerous friends having offered to assist in
the furnishing of the rooms, it will not be long ere the
Farrand nurses are in most comfortable quarters. " The
Duffield Cottage" is another handsome donation re-
ceived by the school to provide isolated accommodation
for the nurses in charge of scarlet fever and diphtheria
patients. We need hardly add that all the fittings in
the Home and the Cottage are of the most modern and
convenient description.
SHORT ITEMS.
A series of lectures on sick nursing are to be given
at Romiley, by Miss Kirkby, in connection with the
the Technical Instruction Committee of the Cheshire
County Council.?Miss Dell is giving a short course of
lectures on sick nursing at Preesall, under the Lanca-
shire Technical Instruction Committee.?A course of
lectures on] the same subject has just been completed
at Wirksworth, they were given by Miss Cooke, under
the auspices of the Derbyshire County Council.?The
nursing staff of the Women's Hospital, Chelsea, has
been augmented, and Sisters and Staff Nurses have
been nominated.?Excellent work has been done by the
Blackpool Ladies Sick Poor Association during the
past year, and the accounts appear to be kept in a
clear and satisfactory manner.?The Ormskirk Local
Government Board have sanctioned the payment of a
subscription of five guineas to the Southport and
Birkdale District Nursing Association.?Last year the
North London Nursing Association sent away one
hundred and fourteen convalescents to various homes
for varying periods.
"THE HOSPITAL" ENDOWED BED.
Under this heading we have chronicled for some
time past the subscriptions which have been sent in
to us towards the annual support of the nurses' bed?
?19 9a. was the total reached last week, including 10s
collected by Nurse Elliott, and ?10 lis. is therefore
still required to make up the ?30 needed for the
current year. We shall be glad to hear from any
amongst our numerous readers who sympathise with
sick and weary nurses. Everyone's thoughts are just
now drifting towards holidays, with a keen realisation
of the fact that all will be better for a little change and
complete rest. It is well to remember that though certain
of our sisters are now, we rejoice to say, receiving fair
and just remuneration for their work, and can afford
to take proper holidays, yet there are a great many
good workers who receive but small wages. Clothes,
shoes, travelling, recreation, and sometimes contribu-
tions towards the support of needy relatives, must
leave actually no balance for the expenses of the holi-
day which temporary indisposition renders an actual
necessity. Thinking of those who are weary and
ailing and poor, surely our readers will gladly make
up the modest sum of ?30 needful to maintain one
free bed for the use of those tired nurses whom we
here represent. All sums sent to the Editor, 428,
Strand, will be acknowledged immediately.
May 20, 1893. THE HOSPITAL NURSING SUPPLEMENT. lxxiii
Sanitation for IRurses.
By a Sanitary Inspector.
VI.-SPAIN.
In Spain wc confess to being specially surprised to find the
laws and regulations for sanitary matters, at least as laid
down by the Government, more complete and enlightened
than might have been expected from the generally backward
condition and the extremely slow rate of advance in that
country [as compared with others on the Continent. But
although the laws are excellent/and the administrative
machinery whereby a board or council for the control of
sanitary matters is provided for every town, is very complete
in its way, nevertheless the people in general reap but little
benefit, and we constantly find epidemics occurring on a large
soale, and but inadequate attempts made tojeffectually check
them. The reason for this unfortunate state of affairs lies
in the fact that the authorities to whom is entrusted the
duty of carrying out the laws and regulations are so
indifferent, and bo little alive to, if not ignorant of, the
great importance of sanitation, that they do as little as they
possibly can, short of being brought unpleasantly to the
notice of the chief authorities in Madrid.
In Spain we find the supreme authority in sanitary matters
is vested in the " Ministre de la Gobernacion," whose duties
correspond as nearly as possible with those of our Home
Secretary, or of the Minister of the Interior in other
countries abroad. Under the control of this Minister is a
Sanitary Council, or equivalent to a kind of Board of
Health," which consists of the Minister himsolf as President,
of a Vice-President, the three Directors-General of Sanitation
?civil, military, and naval?the chief of the navy, a lawyer,
five professors of medicine, three of pharmacy, one of
veterinary science, a civil engineer, architect, and several
others.
In addition to this control and supreme authority, in each
of the various provinces there is a Civil Governor, who has
the direction and control of sanitary matters in his own pro-
vince. He has to Bend in reports at regular intervals to the
chief authority at Madrid.
In the capitals of the various provinces there are what are
called " Provincial Boards " or " Committees of Sanitation,"
of which the Governor of the Province is President, and
these councils supervise all local sanitary arrangements, and
frame regulations for their immediate district.
In every town of over 1,000 inhabitants there is a
"Municipal Board," of which the "Aloalde" is the Pre-
8ident.
Moreover, in every judicial division is a sub-board com-
posed of three members who are called " Sub-delegates of
Sanitation;" one of these is a qualified medical man and
represents medioine and surgery, another member represents
pharmacy, and the third, veterinary science. The "duties of
these sub-delegates are varied and extended. They are
expected to see that all the laws relating to every branch of
sanitation are^thoroughly and efficiently carried out,andinthe
event of an epidemio of infectious disease breaking out in
their division, it is their duty to inform the Alcalde of the
province, who, in his turn, notifies the same to the Civil
Governor, and he I again has to inform the chief I of the
8uprome sanitary authority or council in Madrid. The sub-
delegates have further to inspect chemists' shops at regular
intervals, to superintend the vaccinnation stations in their
division, and are responsible for appointing properly
lualified vaccinators and for seeing that only such persons
ever perform the operation. The sub-delegates further co.
?Perate with the local dootors in all sanitary matters and are
dependent on the latter for information and help of various
kinds. The sub-delegates are appointed and paid by the
Government, and by a Royal Decree of Auguat, 1892, they
were also made sanitary inspectors. By the same decree
several duties were made compulsory which were previously
optional and therefore very often neglected ; amongst others,
that all doctors should be obliged to report to the
sub-delegates every case of cholera or other infectious disease
which came under their notice in the district, which are
further notified to the Alcade, and by him to the higher
authorities. The doctors have further to send in a report)
to the sub-delegates of every case of illness of any kind which
comes under their notice, and to give full particulars of tho
course of the disease, the treatment adopted. They have
also to report all deaths which occur. All those reports are
to be sent in regularly once a week.
In Spain the notification of the following infectious disease!
is made compulsory by law, viz., small-pox, cholera,
diphtheria, scarlet fever, typhoid and typhus, whilst measles
and influenza are not, nor iB consumption notifiable, as it is
in Italy. The notification has to be transmitted, as already
seen, through the various lesser sanitary authorities until it
reaches the Minister.
There are no special hospitals in Spain for isolated cases of
infectious diseases, but in the general hospitals a ward is re*
served for all such patients who are only isolated as far as id
possible under the cirumstances.
When an epidemic breaks out, a special hospital, in Madrid
called the Hospitale Municipale de Vallehermosa, is used
exclusively for such infectious diseases, but the accom-
modation is evidently very insufficient to meet a severe
epidemic, and when there was so much small-pox about
eighteen months ago, the patients had to be placed in beds
along the corridors and were discharged as quickly aB possible,
which, no doubt, greatly increased the number of the
victims. In all towns there is a Hospitalidad Domilicaria,
which provides medical assistance, and supplies food,
medicine, and clothes, when required, gratis to the poor. It is
under the[control of the Alcalde, and the doctors attaohed
to it have to see to the provision of temporary hospitals when
such are required during the time of?'epidemics, and they are
required to give an order, with the consent of the Alcalde,
for the immediate admission of those suffering from infectious
disease, who have no means, so long as the patient or his or
her family give their consent to such removal. If they will
not consent, then the authorities will treat them at their
homes; but this is only allowed if the cases be few, and if
the epidemic spreads, is not permitted under any circum-
stances. In the case of epidemics the utmost power is
granted to the local authorities and boards to do whatever
they consider necessary, and general and local orders are
issued to apply to every case, and at these times at least. are
most rigorously carried out, in addition to the regulations
which are always in force, though not enforced by the
authorities except in times of epidemics.
If a case of infectious disease breaks out in a private
family, the doctor in attendance is supposed to see that the
patient Is properly isolated from the rest of the family, and
if this is not possible he shall have such patient removed to
the special ward of the general hospital. In all cases, how-
ever, the consent of the patient or of his family is necessary
before such removal. The " Ayuntamiento," or Towni
Council, has to appoint an inspector to duly carry out thei
necessary disinfection, which is done free in the case of those
unable to pay, and if any clothing or beddiDg is destroyed
the owner is compensated for the loss at the cost of the
Government.
The usual disinfectants recommended by the authorities
are chloride of lime, sulphurous acid, and dry heat, but it is
very doubtful whether these are efficiently and intelligently
used by the subordinates, when not acting under the imme-
diate direction of their superiors. We have heard of a very
original and wholesale way of disinfection practised on one
occasion in Spain. The hospital, which was full of patients,
some being deliriouB, was surrounded with sulphur, which
was set alight, and had a far from soothing effect on the
nervcB of the sick folks,
lxxiv THE HOSPITAL NURSING SUPPLEMENT. Mat 20, 1893.
Conscientious ^Disinfection.
From a Nurse's Point of View.
Of coarse all disinfeoting ought to be done conscientiously,
just as all drains should be properly constructed, and all
articles of food unadulterated.
In fact, if all classes of workers were true and faithful,
there would be less disease and fewer deaths.
As regards disinfection, for instance, it is by no means
thoroughly understood, and even in experienced hands it
often fails in its object.
Trained nurses certainly cannot be excused for ignorance
on this subject, although much may be forgiven to the pro-
bationer whose experience is necessarily limited.
It is the duty of every nurse to thoroughly acquaint
herself with the objeot of disinfection and the best mode of
carrying it out effectually. When a room has been fumi-
gated, it is the duty of the attendant to know exaotly how
much sulphur has been used, and what preliminary pre-
cautions againBt the escape of the fumes have been taken.
In private houses it is the nurse who is actually responsible
for the whole process, whatever she may fancy. She may
think this duty can be left to others, but it certainly ought
to be entirely supervised by herself, from the first spreading
out of every article to the final closing up of the room.
The doctor and nurse alone know the accurate care re-
quired for each detail, and the latter is certainly to blame if
she shifts her personal responsibility on to others.
Of course, many a mother has learnt by experience and
intelligent observation what disinfection means, but then
numbers of mothers are, ofjcouree, excellent nurses and true
sanitary reformers.
Anyone who owns to not knowing (how much sulphur is
required to fumigatejlarge and small rooms, or says " she isjnot
sure " of the strength of the disinfectant required for soaking
efficaciously the linen of the patient, is certainly no trust-
worthy attendant for cases of infeotious disease.
The lesson is easily learnt, yet if it is not'committed
accurately to memory^it is worse than useless, for it is mis-
leading and dangerous.
Few persons are without some reminiscences of inadequate
disinfection with a disastrous result, and in many cases the
cause has been as evident as the effect.
Of course, our remarks do not apply to the conscientious
woman who is faithful in the smallest detail of her duties.
She is more likely to do too much than too little. But there
are many nurses who are particularly strict in their other
work, and yet^they are quite contented to leave this all.
important point to the discretion of a man who says he
knows all about it. Probably his master may have a com-
plete knowledge of the subject, but he himself has^failed to
grasp fully either the principle or the practioe of it.
Conscientiousness shows particularly in the complete
immersion of infected linen, and the care with which it is
placed in the covered pail on the spot, with no unnecessary
prolonged exposure suoh as must take place when the pail is
kept at a distance.
Even in wards where there is constant supervision of the
workers, there exists great variety in the quality of their
work. A late matron of a children's hospital used to assert
that it was impossible to escape epidemics in such institutions.
This seems to us to savour somewhat of the old-fashioned
theory that pestilences were God's will, than which
surely no doctrine can be more opposed to common-sense
and religion.
Who can read Charles Kingsley's " Two Years Ago " with-
out realising something of the true principles of sanitation ?
He would certainly have said that the lady just alluded to
had failed to grasp even the moBt elementary knowledge of
hygiene.
Stray oases of infectious diseases are constantly imported
into institutions, but when they are allowed to spread, some
one is to blame. There; should be no such factor as chance
allowed for, in dealing with infectious diseases.
To speak more particularly of children, there are a
thousand accidental ways in which the mischief can be done,
and they are none the less dangerous because they are unseen.
When a newly^admitted child is put by a thought-
less nurse to sit on a cot with a little one who has been a
patient for weeks, few people see any risk. The young nurse
thinks herself rather tactful to have hit on a plan for keeping
both her small charges amused and happy. In two or three
days' time the children may be discovered to have developed
similar " rashes," but in the prolonged inconvenience and
regret which ensue, no one probably realises that the second
is a victim to that " want of thought " by which so much
evil is wrought.
If this objection applies to the new patient, how much
greater must it be in the case of little visitors from poor and
insanitary homes ? Again, nothing takes the fancy of the
hospital visitor (always on the look-out for picturesque
effects !) more than a tableau of a number of children grouped
together.
Entering a ward in winter, a blanket is discovered on the
floor in front of the fire, and half-a-dozen convalescent little
ones are seen playing happily together. Truly the picture
is a pretty and often a touching one to thoughtful folks.
But if we put aside sentiment and venture to think for our-
selves, two ideas strike us?firstly, that the floor is not a
good place for children to be transferred to straight from
bed; and secondly, that it is a bad plan to put sick or ailing
creatures into such close proximity. Whilst it can do no
good to one child to have its head close to another little
Bufferer, it is quite possidle that definite harm may result.
In the disinfection of the wards in children's hospitals the
Charge Nurae is certainly quite as responsible as the Matron
and perhapB no better test of a woman's character exists than
this, viz., How will she set to work ? In a medical ward the
process is comparatively simple. Everything can be so easily
spread about and exposed. But a nurse will sometimes shirk
details, or perhaps it is kinder to say, that she forgets that
every article of clean linen stored in her cupboard, each little
thing in her neatly ordered "store" and "stock" shelves
must bo freely opened out to the sulphur fumes presently to
be introduced. Again, she does not always think that every
preparation should be made beforehand to Becure the com-
plete purification aimed at. It is easy to arrange that all linen
in the laundry should be retained there for the moment, and
not returned until the ward can be safely used again, and
this Bhould be the invariable custom. When a surgical or
accident ward needs fumigation, there are more difficulties to
be faced. Perhaps a stock of neatly padded splints lies in
orderly array?pretty little splints, dear to the heart of the
surgical nurse who has stitched away unweariedly, that her
ward appliances should not forfeit the reputation that she has
established for them.
Probably the temptation to believe that no danger can lurk
in these splints is a very great one. If fumigation is suffi-
cient for thick blankets, why not for the splints ? But who
can assert positively that no germs are Becluded in the
closely-packed padding, placed in there with no suspicion of
the infection lurkiug in the ward.
The only safe'rule [when doubt exists is to destroy every
possible source of mischief.
If the nurse be conscientious she will not doubt; her splints
will be unpicked, and padding, covers, wood and iron frames
will be all subjected to the completest disinfection possible*
Every sorap-book, toy, and stray picture will be carefully
May 20, 1893. THE HOSPITAL NURSING SUPPLEMENT. Ixxv
banished, and every corner of the ward will be 30 perfectly
inspected by the nurse's experienced eyes, that her subordi-
nates will have no chance and, we hope, no wish to do their
share of the work less perfectly than she has done hers.
When any inexplicable recurrence of infectious disease
takes place in an institution, it would be well to inquire
"who sees personally to the disinfection?" and "who
inspects all the new cases on admission ?"
These two points being satisfactorily seen to, and inces-
sant watchfulness exercised as to the intercourse between
the children and their outside friends ; the use of separate
towels, cupB, pockethandkerchiefs, &c., being insisted on;
then only will preventable diseases be reduced to a minimum.
If each trained nurse would teach as well as practice, preven-
tive measures thoroughly, and, when required, see that disin-
fection is conscientiously carried out, her duty to her neighbour
would be indeed admirably performed ; and this " duty to our
neighbour " if properly understood, would certainly prevent
the least thoughtful of nurses from transferring to the care
of another, anything out of the ward or room in which a case
of infectious disease has occurred. Precautions are often
carried out intermittently, and the kind-hearted person who
puts a clean garment from her ward linen oupboard on to the
patient she is Bending to an uninfected ward, will perhaps
be quite careful to change her own cuffs and apron, &c.
Consistency and conscientiousness may well go hand in hand.
Everpbo&p'a ?pinion.
[Correspondence on all subject* is invited, but we cannot in any way
be responsible for the opinions expressed by our correspondents. No
communications can be entertained if the name and address of the
correspondent is not given, or unless one side of the paper only be
written on.] -
SANITARY LAW.
"A Practical Nurse " writes : The articles now appear-
ing in The Hospital on " Sanitation " will be doubly valued
by nurses in face of the recent occurrence at Cardiff. It has
hitherto been very difficult for us to get any accurate know-
ledge of the law as affecting the various infectious diseases. I
see that at Cardiff the nurse had to pay ?5 because her ignor-
ance of the Public Health Act had led her to break the law.
I think a careful perusal of your " Sanitation for Nurses "
will Bave your readers from similar blundere.
MEASLES.
"An Ignorant Nurse" writes: I shall be extremely
grateful to any reader of The Hospital who will tell me
whether I should render myself liable to a fine if I accom-
panied a patient suffering from measleB in a cab, without
warning the driver. As measles is not a notifiable disease,
I find a difficulty in getting information. Shall be much
obliged if anyone will tell me.
(Ibe flIMbMesey Ibospital.
An excellent concert in aid of this charity was given by Mr.
Otto Cantor on Monday evening at St. James's Hall. Mr.
Cantor was ably assisted by many well-known artistes,
amongst whom were Mdlle. Jeane Douste de Fortis, Miss
Jester Palliser, Miss Angela Vanbrugh, Mr. Max Reichel,
Mr. Leo Stern, and Mr. BenDaviee. Where the programme
consists of go many excellent items it is difficult to select
any for special remark. Mr. Leo Stern's 'cello solo, Chopin's
Impromptu " in E flat, was most perfectly rendered, and
lollowed as an encore after a graceful song without words by
Mr. Otto Cantor. Mdlle. de Fortis gave a splendid perform-
ance of Liszt's arrangement of Mendelssohn's " Wedding
March." Miss Angela played with grace and artistic feeling ;
Miss Harding's singing and choice of songs were both most
charming, whilst Mr. Ben Davies and Madame Amy Sandon
Were most deservedly popular. There was a fair audience,
amongst which several nurses from the Middlesex were
visible in different parts of the hall. We trust Mr. Cantor's
kind efforts, and those of the ladies and gentlemen who
assisted him, will result in a substantial contribution towards
the hospital.
Metropolitan anb IRaticmal IRursino,
association.
ANNUAL MEETING.
A large number of people assembled at Grosvenor House
on May 15th, to receive the seventeenth annual report of the
Metropolitan and National Nursing Association. The objects
of this Association are as follows :?(1) To train and provide,
a body of skilled nurses to nurse the sick poor at their own.
homes ; (2) to maintain a district home for nurses so trained
to nurse the sick poor in central London; (3) to supply
trained district nursas primarily to the Queen Victoria/
Jubilee Institute for Nurses, and its affiliated associations ^
(4) to raise by all means in its power the standard of nurs-
ing and the social position of nurses.
The Duke of Westminster, President of the Association,
opened the proceedings with a Bhort address, and called
attention to the particularly high character borne by nurses
trained by this Association, and quoted Miss Nightingale's
words that " nursing " means to nurse living bodies and
spirits. The Queen gave ?70,000 from the Jubilee Fund for
the purpose of training nurses for the sick poor, and this-
money, invested in a trust fund, yields an annual income of
?2,000, but this is insufficient to meet the expenses of train-
ing a sufficient number of district nurses to meet the
increasing demand. His Grace then moved, "That the
report be adopted and circulated," and Mr. W. S. Caine,
M.P., seconded the motion, and suggested that probably the.
Queen's Institute owed its very existence to the Metropolitan
Association, for had it not been for the good work done by
the latter from its earliest days, the Queen might never have-
thought of giving ?70,000 for the establishment of the
former. The Rev. A. B. Peile, Warden of St. Katharine's,
supported the motion, and remarked with pleasure on the-
close union between the Queen's Institute and the Metro-
politan Nuring Association. He also Btated that Miss
Hughes, Superintendent of the Metropolitan Nursing Asso-
ciation, would represent the Q.V.J.I. at Chicago, and read-
a paper on its origin and present work. The motion wai
carried unanimously.
The Rev. A. Boyd Carpenter moved ?'That thii
meeting, recognising the good service done by the Metro~.
politan and National Nursing Association, pledges itself to
support the Association in carrying on the work and extend-,
ing the sphere of its operations." He compared the Mrs.
Harris and Sarah Gamp of the past with the splendid women
now engaged in nursing, who demonstrate daily the grand
fact that science and religion can go hand in hand. Dr..
Hawkes supported the motion, and drew attention to the
great work being done in the slums of London by this Asso-
ciation. He deplored the insufficiency of one year's hospital
training for probationers.
The Duke of Westminster being summoned to take his
place in the HouBe of Lords, Sir Dyce Duckworth then took;
the chair. He spoke of the Association as one which
commanded the respect of the medical profession and as the
head of all similar organisations, and he referred to its claims;
and need for increased subscription. The resolution was
submitted, and carried unanimously.
Mr. F. D. Mocatta moved the election of the members of
council for the ensuing year. The Rev. Dacre Craven
seconded the motion, which was submitted, and carried
unanimously.
On the motion of Mr. Bonham Carter a vote of thanks-
was passed to the Duke of Westminster for lending the
room for the meeting, and also for the kind interest he had
constantly shown in the work of the Metropolitan and National
Nursing Association.
Ixxvi THE HOSPITAL NURSING SUPPLEMENT. May 20,1893.
@?t' Eyamtnatlon Questions.
THE NURSING OF BRONCHITIS.
The nursing of bronchitis does not appear to be quite as
popular a subject as the making of poultices and fomenta-
tions. Perhaps there are certain difficulties connected with
this month's question, but surely they would soon disappear
if all our readers would clearly understand that it is simply
on nursing points that we wish them to write.
Bronchitis is no mysterious disease, nor, unhappily, is it
a rare one in the variable climate of the British Isles.
Most probationers have seen it at home, or at any rate
amongst their friends, long before beginning their hospital
training. In fact, we may safely assert that few people have
not some perfunctory knowledge of the requirements of a
case of bronchitis, although possibly it may be with the
chronic," and not the "acute'' variety.
All the answers which have come to us this month appear
to have been written by nurses of considerable experience,
and several of them betoken intelligent personal observation
of the ordinary symptoms and treatment of patients suffering
from acute bronchitis.
Three or four writers have unfortunately lost sight of the
actual question, in their eagerness to suggest treatment. They
forget that answers as to nursing only are desired. Certainly,
if information as to medical treatment were demanded, it
would not be in the pages of " The Nursing Supplement" of
The Hospital.
There exists some little difference of opinion as to the
desirable temperature for the room or ward, one nurse
putting it as low as 60 deg. ; however, 65 deg. is the general
decision, and only one writer wishes 70 deg. to be reached.
An answer from a district nurse gives excellent details,
evincing a clear knowledge of the disease in question, but
unfortunately the stumbling-block, which appears so fatally
attractive to many nurses, involves this writer in an amount
of text-book facts, which would be more appropriate from a
student undergoing a medical examination.
"The patient should be washed daily, on the allotment
system, under a hot blanket," is a delightful sentence, and it
is as descriptive as it is original.
Our nurses are obviously considerate and kindly, for each
one has made some suggestion which proves that her first
thought is for the relief of her patient. Poultices and
pillows are alike ably treated, so as to secure the minimum
of fatigue and the maximum of comfort. Steam kettles and
tencs are carefully described, though some writers forget to
mention the thermometer, which should invariably hang
inside the latter. One only proposes to have no top to the
tent, but we hope this is an unintentional blunder, for we
fear that under such circumstances the amount of steam
which would be retained for the patient's benefit would be
an invisible quantity!
We may also suggest to private and district nurses that it
is always well to explain clearly to the patient's friends the
advantages derived from a moist atmosphere. It is a very com-
mon thing in a cottage to find that the child recently left by
the nuree in a satisfactory environment of steam is removed
from its cot shortly after her departure, because some neigh-
bour feels sure " the poor little lamb" is getting over-
heated.
Even in luxurious nurseries the trained attendant often
has some difficulty in persuading the adults of the household
that the steam must be incessantly maintained, whether she
is absent or present. It is quite common to find an impres-
sion exists that it is good for the patient to allow the moist
atmosphere, which is visibly beneficial, to undergo dangerous
variations of temperature. We give the best answer which
has reached us, and also a question for next month :?
Examination Question for May.
Explain the special nursing required for a case of acute
bronchitis (a) in a hospital; and (b) in a private house ?
Answer.
The nursing of acute bronchitis.
(a) In hospital; (6) In a private house.
(a) One of the great essentials in nuraing a case of bron-
chitis is the maintenance of an even temperature, 65 deg.
being the best height at which to keep the air of the room.
Thisis not always an easy matter in cold weather in a large
hospital ward, so a tent is fixed round the bed in which a
thermometer should hang to gauge the temperature. Steam
is generally ordered for acute cases, as by this means the air
Is not only made warmer, but more moist?moisture is of
great assistance to the patient's breathing. Steam is best
introduced into the air of the tent by means of a long tube
coming from a kettle boiling over a spirit lamp placed on a firm
table by the bedside. Kettles of this kind are in general use in
most hospitals. Care should be taken that any outward appli-
cation ordered,{such as poultices or fomentations, should be
light, and changed before getting cold, as a cold, heavy
poultice is worse than nothing at all. They should be
placed well over the entire surface of the chest, quite up to
the collar bones. Heart complications often ensue in
bronchitis cases, especially in stout and elderly subjects, so
such signs as lividity of face, sudden increase of difficult
breathing, or sometimes stoppage of breath altogether,
should be watched for, and immediately reported to the
doctor. In cases where a doctor is not at hand, a mustard
plaster over the heart and an ounce of brandy in warm water
can be given with advantage, and artificial respiration used
in extreme cases. If the change proceeds from choking an
emetic could be resorted to. The patient's temperature,
pulse, and respiration should be taken night and morning
(oftener if desired by the doctor), and the sputum saved for
inspection. The state of the bowels, skin, appetite, cough,
and amount of food and sleep taken in the twenty-four hours
should be noticed to report to the doctor. A light diet of
milk, beef tea, eggs, fish, &c., is generally ordered, and
should be given in small quantities frequently, and so
arranged as not to clash with stimulants and medicines.
Bronchitis cases require plenty of pillows or a bed-rest to
raise the head and shoulders, as lying quite flat adds much
to the difficulty of breathing.
(b) To nurse a private case of bronchitis an airy room with
a sunny aspect and open fireplaoe should be chosen. A small
spring bed with a good hair mattress, plenty of pillowB, and
light warm clothing is the best couch for the patient.
Curtains at the head of the bed and over the door are useful
to exclude draughts. The bed should not be very far from
the fireplace, so that if steam is required and has to come
from a kettle on the fire its benefits will be greater. In the
absence of a regular spirit-lamp kettle an ordinary tin one
holding four quarts can be easily converted by a tinman into
a bronohitis kettle by having the usual spout soldered up and
one made at the top on to which is fitted a tin tube three or
four feet long to conduct the steam into the room. Lamps
or candles are the best lights, as gas dries the air and
increases the cough. The thermometer must be consulted
frequently to ascertain the temperature of the room, which
should Btand at 65 deg, Mt?ny visitors are undesirable aa
cold air enters with them, and they exhaust the
patient by talking. A chair placed upside down at
the back of the patient, well padded with pillows,
makes a good bed rest. A private nurse ought to be
able to think out dainty dishes for her patients. Notes of
temperature, pulse respiration, state of bowels, skin, &c.,
should be kept for the doctor's visit. Cold feet will often
make the breathing worse. When a convalescent patient is
allowed to leave the sick room, chills in passages Bhould be
guarded against, and the room he goes into must be no
colder than the one he leaves. A respirator or woollen scarf
Bhould be worn over the mouth on first going out of doors.
G. M. Payne.
Examination Question for June.
What are a nurse's duties with regard to the prevention
and treatment of bedsores ?
Answers must be ssnt in by June 5th. They must be
clear and concise, written on one side of the paper only, ac-
companied by writer's name and address, and directed to
"Nursing," Editor of The Hospital.
Mat 20, 1893. THE HOSPITAL NURSING SUPPLEMENT. lxxvii
appointments.
[It is requested that successful candidates will send a copy of their
applications and testimonials, with date of election; to The Editor,
Tbe Lodge, Porchester Square, W.]
Hospital of St. Cross, Rugby.?Miss Heathcote has
been made Matron of the Hospital ?of St. Cross. She was
trained at Pendlebury, and at St. Bartholomew's Hospital,
and afterwards worked at the Western General^Dispensary,
London, and we congratulate her on her present appointment.
North Cambridge Hospital.?Miss Emily Stendell has
been appointed Matron of the North Cambridge Hospital.
Miss Stendell was trained at Leeds Infirmary, and was after-
wards a sister at Bristol Children's Hospital, and subsequently
Matron of Taunton Sanitary Hospital. Miss Stendell takes
many good wishes with her to her fresh work.
Sanatorium at Repton School.?Miss Julia Birmingham,
for some years Sister at the Royal Hante County Hospital,
Winchester, has been appointed Matron of the Sanatorium
at Repton School. Miss Birmingham was Matron for some
time of the Bickiey Cottage Hospital.
Victoria Hospital for Sick Children, Hull.?Miss
E. T. Batchelor, who trained at St. Thomas's Hospital, has
been appointed Matron of the Victoria Children's Hospital,
Hull. Miss Batchelor was for three years at the County
Hospital, York, and afterwards at Cumberland Infirmary ;
Royal Infirmary, Preston ; Sanitary Hospital, Bournemouth ;
and Small-pox Hospital, Sheffield. Such valuable and varied
experience must render Miss Batchelor peculiarly fitted for
her new post, in which we wish her every success.
presentation.
On the third anniversary of the opening of the Home and
Hospital for Jewish Incurables at Victoria Park the Com-
mittee presented the two head nurses each with a handsome
gold watch. These bore inscriptions to the effect that they
were given as marks^of esteem to Sister Anstey and Sister
Davis, who have been associated with the hospital since its
commencement.
"Motes an& ?ueries.
special notice.
The contents of the Editor's Letter-box have now reached such un-
wieldy proportions that it has become necessary to establish a hard and
fast rule regarding Answers to Correspondents. In future, all questions
requiring replies will continue to be answered in this column without
any fee. If an answer is required by letter, a fee of half-a-crown must
be enclosed with the note containing the enquiry. We are always
pleased to help our numerous correspondents to the fullest extent, and
we can trust them to sympathise in the overwhelming amount of
writing which makes the new rules a necessity.
Queries.
(128) Address Wantei.?Can you give mo address of St. John's
Ambulance Ascociation. as I am in a remote country district where
thero is m local branch ??Malc Nurie.
(129i St. Mark's Hospital.?Where is this hospital situated in London ?
?W. B.
(130) Elizabeth Jones.?Where can I get the Life of the later Miss
Elizabeth Jones ??Sandy.
(131) Probationer.?Please tell me how I can got a berth as proba-
tioner, and is 19 too young to begin ??A. de M.
(132) Couch.?Can you advite me as to the best couch and operating
table for small surgical homo P?Liverpudlian.
(133) Creches.?At what crCohes could a lady get training ? A small
premium could bo paid if nooes?ary.?Nemo.
(134) Training.?I find it difficult to hear of a vacancy in a general
Elcan*^ *?r a Pro^ationer' "Where would you advise me to try next ??
Answers.
(128) Address Wanted (Male Nurse).?St. John's Ambulance!Afisocia-
Gate, Cltrkenwell, E.G.
(1,29)i St. Marie's Hospital IW. If,).?See Burdett's " Hospital Annual,"
Scientific Pres., 428. Strand.
(ISO) Elizabeth Jones (Sandy).?"Memorials of Agnes Elizabeth
H^U^Lond^ k?r a'Btor* Published by Htrahan and Co., 53. Ludgate
(131) Probationer (A. de JVf.).?Yes, quite too youDg. Read " How to
^nrso-" by Honner Morten, prioe 2s. 6d.. 428, Ntrand.
tti T" Couch (Liverpudlian).?You Bhould go and see what is used in
"o Liverpool hospitals and infirmaries, and write to the maker of the
?ne you like best. We saw ?n excellent operating table at the Cromer
uottage Hospital, which had been made by a local man at a most mode-
rate price. Matrons and secretaries are always willing to give informa-
tion on practical pointr.
033) Ci cchts (Nemo) .?If you write to the Matron or Head Nurso of a
creche situated in the district where you desire to go, you will get your
question answered immediately.
(134) Training (Eleanor).?Certainly, you should write or apply to the
Hon. Secretary of the Workhouse Nursing Association, 6, Adam
otieet.W.C.
fov TReaMng to tbe QkK
When travellers speak of the way they are going, their
words do not so much suggest the road, as the end, to which
they are tending. They have had to choose their route, as
every path leads somewhere, either in the right direction or
in the wrong. Now every man, wcman, and child is
travelling towards an end to which there are two distinct
roads, the broad, and the narrow road, which have very
different terminations. The season of sickness is a good
time for ub to pause and ask ourselves, along which are we
going ? We started, perhaps, heedlessly, without due con-
sideration, or had even determined to take the more difficult
path, but the broad road looked so sunny and cheerful, so
full of pleasure and amusements, that we soon strayed into
it, and went farther and farther, until it became a wilder-
ness in which we were altogether lost. Aimlessly we
stumbled on, sometimes over great stones, which brought us
rolling to the ground, tearing our clothes with briars and
thorns, soiling our feet with mire and dirt, which we had not
noticed, or taken no trouble to avoid. Oh ! the unhappiness
we have caused our friends by our recklessness ! Oh ! the
sorrow and misery we have brought on ourselves ! and what
will be the end thereof, unless we turn our steps into the
narrow path ? But now we cannot find the way, and cry in
the bitterness of our hearts, who will show us the way which
leadeth to eternal life ? Christ, in His great love, answers
us, " I am the way, the truth, and the life." I am the way,
it is the truth I am telling you, and I will lead you to the
eternal mansions prepared by your heavenly Father for those
who love Him. Though our dear Lord has gone far
from the sight of mortal eyes, yet He has promised
to come again, and take us to Himself. But ere that time
arrive He will comfort the repentant soul with His choicest
blessings, for He says, " Though your sins be as scarlet, they
shall be white as snow ; though they be crimson, yet shall they
become Iike"wool." And with Christ's redeeming power to
help us, we must watch for His coming. It may be in the
morning or the evening, or at cockcrow; but the hour when He
comes will be the hour of our death. Let us walk warily, fov
at the end of our journey there is a stream, narrow, but im-
passable without the help of Him Who has promised to be
with us. We stand and shiver at the brink, for we forget
His words, "I am the way." His boundless love has made
a bridge, oyer which our feeble trembling steps may tread
safely. His blessed Body, nailed to the Cross, stretches
across the dark river which divides earth from heaven, and
we shall say, each one, as we clasp the Master's hand?
The great and terrible land
Of wilderness and drought
Dies in the shadows behind me,
For the Lord hath brought me out.
The great and terrible river
* * * *
Lies in the shadows before me?
But the Lord will bear me through.
IRurstng at Southampton.
The Queen's Nurses are doing excellent work at South-
ampton, and the care of the sick poor in that town is for the
future to be left entirely in their hands. The Hampshire
Nurses' Institute, which for many years undertook to supply
trained nurses for those who can pay, and also for the poor,
have now relinquished the latter and arranged to provido
skilled attendants for the former class of patients only.
TKHants an& TOlorftcrs.
Can anyone give the Address of Princes3 Beatrice's Nwrting Guild
to a Monthly JJurso P
W ill anyone kindly give an old hip carriage for tha use of a tiny hoy
in the Melicent Borne,Sandowa I tie of Wight P
lxxviii THE HOSPITAL NURSING SUPPLEMENT. Mat 20, 1893.
XTbe nouses' Xooftmcj Glass.
PICTURES.?THE ROYAL ACADEMY.
When people intend to visit but one of the summer
collection of pictures, the choice almost invariably falls on
the Academy. Yet the Academy proves a serious under-
taking for a summer's day even for the strongest of ub. If
we really spend a reasonable amount of time over the
pictures, Academy headache is almost sure to be the result.
This year the hot weather coming so early has greatly added
to the number of the victims, and the stifling atmosphere of
the crowded rooms must materially, if unconsciously, have
influenced many a criticism passed.
Those who look for one or two pictures standing strikingly
apart from the rest as a sine qua non of an Academy exhibi-
tion will experience disappointment. For several years
now there has been an absence of these planets amongst
the lesser stars. This year it would be harder than ever to
state the particular pictures which have fixed the public
fancy, although there ia a large contingent of admirable
works shown. There are, as usual, plenty of portraits ;
good, bad, and indifferent. Some of them are of remarkably
plain people. Four Royalties are represented, an unusual
feature in the portraits of the year. The Duke of Cambridge
and the Prince of Wales occupy opposite corners in the
same room. Both portraits are life-size, and ably repre-
sent their Royal Highnesses. The Prince of Wales, in
levee dress, seems as if he were about to Btep out of the
picture and dance a minuet. A portrait of Sarah
Bernhardt will interest many. It is a beautiful pro-
duction iu every way, and a most pleasing representation of
the great French actress. It is a half-length portrait, nearly
in profile. She is seated, enveloped in a beautiful white
cloak, her splendid Venetian red hair showiug to the utmost
advantage. The whole portrait is powerful and dignified.
The Duke of Devonshire, Sir James Sigall, Princess Ferdi-
nand of Roumania, Lord Tragner. Colonel Bernardiston, the
Hon. H. Fowler, M.P., Joachim, Lord Rockwood, Lady Ada
Osborne, Dr. Bristowe, Lady Blomfield, Sir Henry Parkea,
Miss Margaret Balfour, Lady Margaret Sackville, the
Countess Fitzwilliam, Lady Dorothy Neville, Sir Cfiarles
Tennant, are portrayed by various artists.
During our visit to the Academy we were amused to hear
conflicting opinions as to portrait* wiuhin the apace of five
minutes. One spectator observed, " I never cire for the por-
traits,"whilstanother remarked that the only kind of pictures
that he really found interesting were the portraits. Diverse
as may be the taste for and against this branch of pictorial
art it is so varied in the present exhibition that the portrait
is often quite as much a picture as a subject painting could
be. Witness, for instance, the portraits of Lady Blomfield,
Miss Hej?an Kennard, and Lady Agnew, and the one en-
titled "The Qaeen of Love."
Many artists have made happy choice of interesting
subjects this year for their pictures. Quite one of the
most striking of these is Frank Dicksea's "Funeral of
a Viking." Out into the waves is being launched the
barque on which is lain the white-bearded warrior, his
face wearing a reposeful and majestic express ion in death.
He is in full armour. By his side rests his shield ; his sword
is grasped in his right hand. The wave which receives this
funeral pyre is crimson with the burning mass, and from the
flame of the torches which lighted it, still burning in the
hands of the assistants at the ceremony. These stand in a
group at the edge of the wave, with faces alight with the
inspiration of the moment, their armour gleamiDg in the
glowing red which illumines the whole scene, save the
gloomy background of high cliffs. A sea bird flies bewildered
lound the scene.
A direct contrast to this picture is Miss Margaret DickHee'a
"The Child Handel." This is a most loveable picture.
The small child Handel, in his little night dress and night
cap, is seated on a high chair, his little bare feet some
distance from the floor, playing at a spinet. History says
that the small child had frequented the garret where the
spinet stood at nightfall, a practice that ended in the dis-
covery portrayed in the picture. First comes the father
with his lantern held high, shedding its full light on
little Handel's figure ; behind the elder Handel comes a
group of half-alarmed, half-curious spectators. The
picture is, to our mind, one of the most pleasing of
this year's exhibits. Another interesting composition is
"The Duchess of Richmond's Ball at Brussels on the Eve
of the Battle of Waterloo." The second messenger from
Bliicher is arriving, and a general air of expectancy is
visible throughout the room.
One of the most remarkable pieces of colouring in the
Academy is Solomon Solomon's picture, which bears the title-
" Your Health." The merit of the picture lies in the
atmosphere of colour it conveys. Mr. Solomon has grouped
a number of his friends at a dinner table, and they are in the
attitude of drinking the health of the host. The softest rose-
pink light falls on all from rose-shaded lamps. The table is
decorated with roses, and the dresses of the ladies lend them-
selves to the soft tones and catch the colour* in gleaming
folds of white. Thus a purely material subjeot has been used
to produce a most poetic effect. We have been so used to Mr.
Solomon's classic subjects that a picture such as-
this from the same brush is quite a revelation.
It affords a diatinct pleasure to many a visitor to picture
exhibitions if they can name the artist by a glance at the
picture. It is to be doubted if it is really a compliment*
to the artist to be able thus to detect his work
at a glance, as it is usually some trick of mannerism,
than intrinsic merit of uny kind which is the trade
mark so familiar to the passing observer. In this dinner-
party scene of Mr. Solomon's the painter has certainly
hidden himself.
There are several other interiors where the hour
of meals has been chosen for portrayal, the most
interesting of these being that of a party of children. The
picture is left to tell its own story, as it! is simply
entitled " Evening : Children Saying Grace." The-
room in which this picture hangs (No. 10) seems especially
devoted to the little ones. In one corner hangs a touching
little picture (803) called " Grandfather's Little Nurse."
The tender way in which the little girl is waiting on.
her old grandfather is very prettily given.
One picture that calls forth a great deal of attention
is "The Announcement." The full meaning of the
picture is open to question. By the fire sits an old
woman; in the centre of the room, half shrinking
back, stands a sorrowful faced woman in widow's dress ?
a third figure, that of a working woman, leans over the old
woman evidently acquainting her with the presence of the
widow. The firelight plays on the face of the old lady,,
and through her withered hands. A lamp on the table is-
shedding its light on the tea things, whilst daylight-
is still trying to assert i' self through the cur-
tained window. The accessories in this picture, and
the effect of the fire and lamp light, are wonderfully
depicted, and the picture is altogether a very interesting one;
Charity iB represented by a picture of " Hospital Sunday "?
the collection is being made in a village church ; not a very
interesting picture ; and by "The Orphansdemure little
maidens in quaint caps and gowns, seated on forms, all
diligently sewing?a picture that gives a sadly subdued
phase of child life; and lastly by a "Nurse from the
Children's Hospital" holding in her arms the poor little
baby charge. There now remains but landscapes to be noted.
An English exhibition always contains a very large propor-
tion of these, as so many of our artists devote them-
selves to landscape painting. Room No. 1 introduces us to a
number of charmiDg scenes. Leader contributes two sunny
studies to this collection, Nos. 3 and 39. David Murray's-
"Meadow Sweets," in quieter tone, is more refreshing to
the sight on a hot day. Tie soft grey-green of the hayfield
and belt of willows is Nature speaking through canvas.
"Fire Faggotp," by the same artist, is a warmer picture of
heather land, amorgst which the tired faggot gatherers are
lyiDg; beautiful rich dark green treeland stretches to the
confines of the landscape. There are several tumultuous
stretches of solitary blue seas?ao fascinating to the painter?
and a wonderfully pre-Raphaelite study of a country lane
(103), which is bordered with bushes of roses and elder,,
drawn with great exactness and brilliancy. A nurse-
spectator much admired this certainly remarkable pro-
duction, but her friend, who appeared to pose as a " critic,"
considered her taste far from orthodox, and Baid she had
hoped " better things of her." For our part we like to be
allowed to like what we do like, though it may not be what)
artistic regulations say we should, and so we have a fellow
feeling with the chidden nurse. From amongst the 1 22?
oil and water colour paintings there must be something for
all tastes, and in drawing attention to any of the pictures
in particular we have to keep to very broad and general
lines, hoping that to most will come the opportunity of
seeing and judging for themselves.
May 20,1893. THE HOSPITAL NURSING SUPPLEMENT. lxxix
Zbe 3Book Morlb for Women aitfc IFlursee.
tWe invito Correspondence, Criticism, Enquiries, and Note' oil Books likely to interest Women and Nurses. Address, Editor, The Hospital
(Nurses' Book World), 428, Strand, W.O.I
THE MAY REVIEWS.
In the Nineteenth Century Canon Browne discussea the
Higher Education of Women with an eye to the future. Few
men have had more varied and long-continued experience in
the examination of men and women, or better opportunities
of weighing the results of a given course of study. It is
Therefore a matter of some interest to learn that he is him-
self " opposed to the conversion of Oxford and Cambridge
into mixed Universities for men and women, and to the
opinion that the higher education of men and the higher
education of women should be identical." Not for a moment,
however, must it be supposed that Canon Browne is an
advocate for cramping the latter. What he urges is a scheme
for its fuller development, unhampered by the complicated
systems which experience and compromise together have
established at Oxford and Cambridge. He proposes the
establishment of a central council, endowed by Royal
Charter, with power to grant degrees to women ; in brief,
the establishment of an Imperial University for women, with
fellowships and professorships, and due provision for " sub-
jects specially suited to women's intellects, and, above all,
to women's wants."
"Good and Bad Mothers" is a pretest by Mrs. Amelia
Barr in the North American Review less against bad
mothers than bad nurses. It appears that the nursing out
eystem is in full force in the States. '* The child of the
fashionable woman is too often committed at once to the
care of some stranger who, for a few dollars a month, is
expected to perform th? mother's duty for her. What these
neglected little ones suffer from thirst is a matter too painful
to inquire into. Many a baby, after beiDg tortured for hours
with a feverish, consuming thirst, passes into the doctor's
hands before the trouble is recognised." Mrs. Barr also com-
ments on the miseries endured by these little nurse children
intheparks, "where the hot sun beats down on the small
upturned face, and the child is at shrieking point from lying
too long in one position." The evils at which she glances to
which the young girls are exposed in the discussion before
them of "questionabl e subjects" are of an even graver
nature.
It is instructive after this to read the contrast drawn in
Macmillan on the education of girls in this country now and
fortyyears ago. To the child of bygone years the devil was an
ever present reality. " Nurse," she would say when put to
bed,trembling in every limb, " come and sit beside me. The
devil is Bitting grinning at me in that corner ; do you know
what he's tempting me to do ? " " No, my lamb ; what ? "
the person addressed would reply sympathetically. " He's
tempting me to pray to him." " Ob, shocking ! " says
nurse. On the next night the same scene would be recom-
menced. " Nurse, I'm tempted again. " I'm tempted to
pray to the Virgin Mary." " Oh ! horrible, frightful," says
nurse, this latter temptation occurring to her as infinitely
the more shocking of the two. Clearly, Patty should have
Ixxx THE HOSPITAL NURSING SUPPLEMENT. Mat 20, 1893.
THE BOOK WORLD FOR WOMEN AND NTJRSES-conrtnuei.
been taken to a doctor for her temptations, and ordered a
course of cod-liver oil for her dreams, as the writer suggests.
But the modern child is, perhaps, over-sheltered in this
present world as well as from all terrors of the future.
" Wh8n it becomes necessary to extract one of her small teeth
gas is administered, for it would never do for her to suffer the
least pain. She is swathed up to her neck and wrists in
lambs'-wool, and would have stared at poor Patty's shore
sleeves and rough, red arms. No morbid fancies afflict
her, if they did she would promptly be taken to see the
family doctor. If she is naughty it means that she is
' below par,' and must take a tonic." The reason of the con-
trast is really not far to seek. It is that the unhappy little
Patties have turned into the modern mothers, and have vowed
that their children shall never suffer as they did. What new
sort of mother will the modern " soft system " develop in its
turn ?
MAGAZINES FOR MAY.
The Cornhill Magazine.?This number of the Cornhill
contains an interesting article on "Needle Craft." The
title, needle craft, gives the writer an opportunity of
treating the subject with a dignity above the consideration
of crewel work, antimacassars, wool flowers, and the
like; consequently, modern needlework does not come
within the range of the article. The author seems to
place the decay of needle craft as contemporary with
the death of the sampler. We ourselves would place it at
an earlier period, and argue that its decay as a craft was
gradual, and parallel with the higher mental culture of women.
Those yards and yards of wonderful historical tapestry could
not have been worked by those whose whole mind and time
were not given to the subjesb. The long tedious hours had to
be filled somehow, and filled they were with rigorous
regularity in the production of the miles of minutely
fashioned needlework which remain to astonish modern eyes
with the patient industry of the past. Tapestry gave the
workers at least an incentive to labour at the needle, as,
when finished, it was a veritable piece of workmanship ; and
perhaps still more so was the applique work which came in
later, and which formed gorgeous Court trains, hangings, &c.
Such work was a necessary part of the daily life of ladies
in society, and the writer of " Needle Craft" quotes of
Mary Queen of Scots that " All day she wrought
with her nydilli and that diversity of the colours
made the work less tedious, and she continued so
long at it till very payne made her give over." In poor
Mary's case needlework proved unequal to satisfy her and
keep her from mischief. To no one of active body or brains
could needlework prove a satisfying occupation, and although
we may admire the work of past times, and the really
beautiful art needlework wrought by the few in presenf;
times, we cannot share the regret expressed occasionally that
needlework as an art is fast dying out from female educa-
tion ; but we regret that a little more incentive is not given
to the use of the needle for homely purposes. No girl is
the worse for knowing how to make her own olothes, while
the want of this knowledge debars many a girl from enjoy-
ing her life as Bhe might, and although our home made gar-
ments may never become historical, or be worthy of pagps
devoted to their description in future numbers of the Gornhiil,
they may have helped to wile away hours in just as pleasann
a manner, and be regarded with pride equal to that felt by
mediceval ladies in a needle-wrought castle.

				

